# Nanoscale 2.5-dimensional surface patterning with plasmonic lithography

**DOI:** 10.1038/s41598-017-10047-0

**Published:** 2017-08-29

**Authors:** Howon Jung, Changhoon Park, Seonghyeon Oh, Jae W. Hahn

**Affiliations:** Nano Photonics Laboratory, School of Mechanical Engineering, Yonsei University, 50 Yonsei-ro, Seodaemun-gu, Seoul, 120-749 Republic of Korea

## Abstract

We report an extension of plasmonic lithography to nanoscale 2.5-dimensional (2.5D) surface patterning. To obtain the impulse response of a plasmonic lithography system, we described the field distribution of a point dipole source generated by a metallic ridge aperture with a theoretical model using the concepts of quasi-spherical waves and surface plasmon–polaritons. We performed deconvolution to construct an exposure map of a target shape for patterning. For practical applications, we fabricated several nanoscale and microscale structures, such as a cone, microlens array, nanoneedle, and a multiscale structure using the plasmonic lithography system. We verified the possibility of applying plasmonic lithography to multiscale structuring from a few tens of nanometres to a few micrometres in the lateral dimension. We obtained a root-mean-square error of 4.7 nm between the target shape and the patterned shape, and a surface roughness of 11.5 nm.

## Introduction

The development of three-dimensional (3D) micro- and nanofabrication has attracted a lot of attention because of its relevance to various fields^1–6^. Fabrication of complex micro- and nanostructures has been demonstrated with several 2.5D surface patterning technologies, such as electron-beam lithography (EBL), laser direct writing, grey-scale lithography, two-photon polymerisation lithography, focused ion-beam (FIB) milling, and thermal-probe-based patterning^7–12^. Nevertheless, in terms of practical applications, scalable and cost-effective fabrication methods for nanostructuring are in the early stages of development.

Although the above patterning techniques can produce high-resolution features on resist materials, the thickness of the resist material should be limited in order to achieve high resolution, because of the electron-beam broadening and ion scattering in the e-beam and FIB lithography techniques, respectively^13, 14^. As for probe-based patterning techniques, the resolution and pattern shaping are determined by the shape of the tip apex, and high-resolution patterning processes with smaller pixel sizes could be extremely time consuming. 2.5-dimensional surface patterning with molecular resist could suffer from contamination of the process environment due to the direct chemical process used to remove the resist with a thermal probe^10, 11^. Two-photon polymerisation lithography uses focusing laser beams with precise control of pulse power for fabrication of micro/nanostructures. Therefore, there is still a need for scalability from the nanoscale to the microscale and cost-effectiveness of fabrication methods, which can be adapted with the various lithography techniques offering geometric versatility of target objects.

Plasmonic lithography has been studied to extend its potential application to nanoscale fabrication^15–18^. Because of its simpler system and low-cost process, it has been regarded as one of the significant candidates for nanofabrication techniques^15–19^. By employing a sub-diffraction-limited size spot generated by a metal ridge aperture, which has a high cut-off wavelength, plasmonic lithography can be used to obtain a resolution of 22 nm in half-pitch^19, 20^. Research on plasmonic lithography has been focused on achieving higher resolution and throughput. In order to extend the scope of plasmonic lithography to nanoscale 2.5D surface patterning, we need an exact understanding of the distribution of the electromagnetic field emitted from the aperture in terms of surface waves and space waves.

In a single aperture, as the excitation efficiency of surface plasmon–polaritons (SPPs) is directly related to the wavelength of light and the geometrical features of the aperture, diffracted light without SPPs also contributes to transmission in the form of quasi-cylindrical waves (QCWs) in the slit aperture^21–24^. Recently, the concept of spherical waves (QSWs)^25^ was proposed on the same lines as QCWs in the slit aperture, because a hole-type ridge aperture radiates spherical-like creeping waves. For a ridge aperture, the incident magnetic field induces oscillation of accumulated charges at the side of the ridges; each oscillating charge acts as a point dipole source, called a localised surface plasmon (LSP)^26, 27^. In the ridge aperture, surface waves from the point source and LSP are composed of SPPs and QSWs, and the field intensity distribution of light transmitted through a bowtie aperture have been successfully described in analytical form on the basis of SPPs and QSWs^25^.

In this work, we extend the feasibility of applying the plasmonic lithography technique to nano–micro 2.5D surface patterning. To obtain an exposure map for the plasmonic lithography system, we adopt an analytical model to exactly describe the field intensity distribution of light transmitted through a nano-aperture in a metal film. We develop a pattern-prediction algorithm to calculate an exposure map in terms of pixel position and exposure dose to optimise the surface patterning process of a target pattern. The performance of plasmonic lithography in surface patterning is investigated by patterning various nanostructures, such as a microlens array (MLA), nanoneedle, and multiscale nanostructure. In addition, the fabrication error and surface quality of the patterns are quantitatively analysed.

## Results and Discussion

### Description of impulse response of plasmonic lithography system

**Figure 1:**
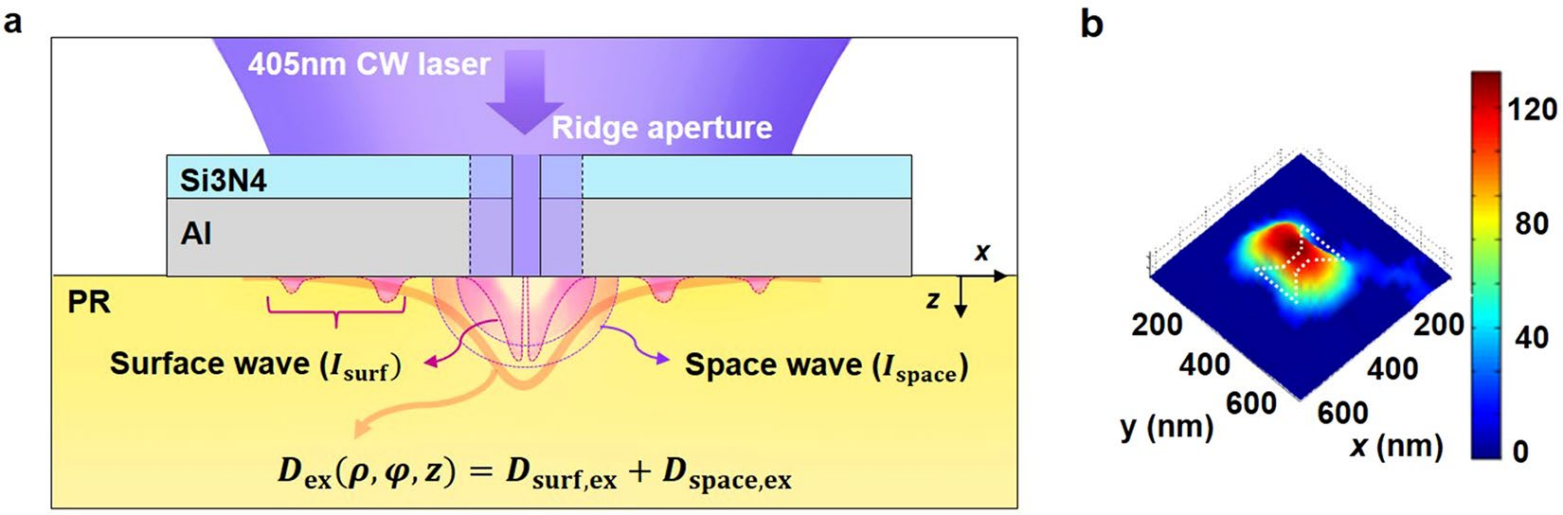
Schematic of transmitted field distribution excited by a ridge aperture and an exposed spot pattern. (**a**) Schematic of field distribution generated by a metal/dielectric ridge nano-aperture for 2.5D surface patterning by plasmonic lithography. *D*_surf,ex_ and *D*_space,ex_ are the dose distributions from the ridge aperture obtained by the surface wave and space wave, respectively. The spot pattern matches the total dose distribution *D*_ex_. (**b**) Typical spot pattern recorded with the plasmonic lithography system using a bowtie aperture.

As light is illuminated on a bowtie aperture, the LSPs resulting from interaction at the metallo-dielectric interface and transmitted field through the aperture generate field distributions around the aperture. The LSP excites surfaces waves, which consist of SPPs and QSWs; the surface waves have an azimuthal angle dependence owing to the dipole characteristic. On the other hand, owing to the small size of the 2D hole, diffraction of the transmitted fields can be approximated as radiation by a point source. In previous research, the 2.5D field distribution in a ridge nano-aperture was verified with an analytical formula^25^. A schematic of the transient field distribution from a bowtie-shaped ridge aperture for plasmonic lithography is described in Fig. 1.

The transmitted field described by the point source propagates spherically from the exit plane of the aperture, which is called a space wave. As the photoresist (PR) is illuminated by light, the exposure dose, *D*_ex_, can be defined by *D*_ex_ = *It*, where *I* is the total intensity of the field, and *t* is the exposure time. The profile of the spot pattern matches the distribution where *D*_ex_ is equal to the threshold dose of the PR, *D*_th_. The total local exposure dose, *D*_total,ex_(*ρ, φ, z*), can thus be described by the summation of *D*_surf,ex_(*ρ, φ, z*) and *D*_space,ex_(*ρ, φ, z*), where *ρ, φ*, and *z* are parameters for cylindrical coordinates. We can specify the transient exposure dose profile of the spot pattern with an analytical function as follows:^25^

1 $${D}_{\mathrm{total},\mathrm{ex}}(\rho ,\phi ,z)=\{\begin{array}{l}{I}_{0,{\rm{surf}}}t\exp (-\frac{{(\rho -g/2)}^{2}}{2{{\sigma }_{x}}^{2}})\exp (-\alpha z){\cos }^{2}\phi +\frac{{I}_{0,{\rm{space}}}t}{{z}^{2}}\exp (-\frac{{\rho }^{2}}{2{{\sigma }_{y}}^{2}})\,\,\,{\rm{for}}\,\rho \ge g/2\\ \frac{{I}_{0,{\rm{space}}}t}{{z}^{2}}\exp (-\frac{{\rho }^{2}}{2{{\sigma }_{y}}^{2}})\,\,\,\,\,\,\,\,\,\,\,\,\,\,\,\,\,\,\,\,\,\,\,\,\,\,\,\,\,\,\,\,\,\,\,\,\,\,\,\,\,\,\,\,\,\,\,\,\,\,\,\,\,\,\,\,\,\,\,\,\,\,\,\,\,\,\,\,\,\,\,\,\,{\rm{for}}\,\rho  < g/2\end{array},$$

where *α* is the attenuation coefficient due to the normal wave vector of the surface wave; *g* is the gap distance in the ridge aperture; *σ*_*x*_ is ${w}_{x}/2\sqrt{2\,\mathrm{ln}\,10}$, where *w*_*x*_ is the full width at tenth maximum (FWTM) in the *x*-direction; *σ*_*y*_ is ${w}_{y}/2\sqrt{2\,\mathrm{ln}\,2}$, where *w*_*y*_ is the full width at half maximum (FWHM) in the *y* direction. *I*_0,surf_ and *I*_0,space_ are the peak intensities of the surface wave and space wave, respectively. Unlike the previous literature, we revised the theoretical model by limiting the near field to the walls of the ridge side without a change in the calibration curve. Unlike the equations previously studied, a revised equation is introduced to reflect the characteristics of the near-field distribution, which is confined on the metallic surface. By imposing the threshold dose of the PR on the left-hand side of equation (1), the pattern distribution generated by light exposure can be obtained. Geometrical parameters such as *w*_*x*_ and *w*_*y*_ are dependent on the laser intensity and exposure time, and we determined the parameters in the analytical formula by a calibration process using spot patterns exposed by the plasmonic lithography system. As described above, the dimension of the x-width is determined by both the propagating field and the evanescent field induced by plasmonic interaction. However, because the field distribution around the metallic surface is strongly affected by plasmonic interaction, the FWTM located near the metallic surface is used for point spread function (PSF) modelling in the x-direction. The parameters of the analytical formula used for the patterning are listed in the Experimental section.

### Calculation of exposure map for plasmonic lithography

If we decide that a target shape needs to be recorded on the PR, an exposure map is necessary to operate the plasmonic lithography system. This exposure map consists of the exposure location, laser intensity, and exposure time. As the target shape is determined, the exposure map can be obtained with a deconvolution procedure.

**Figure 2:**
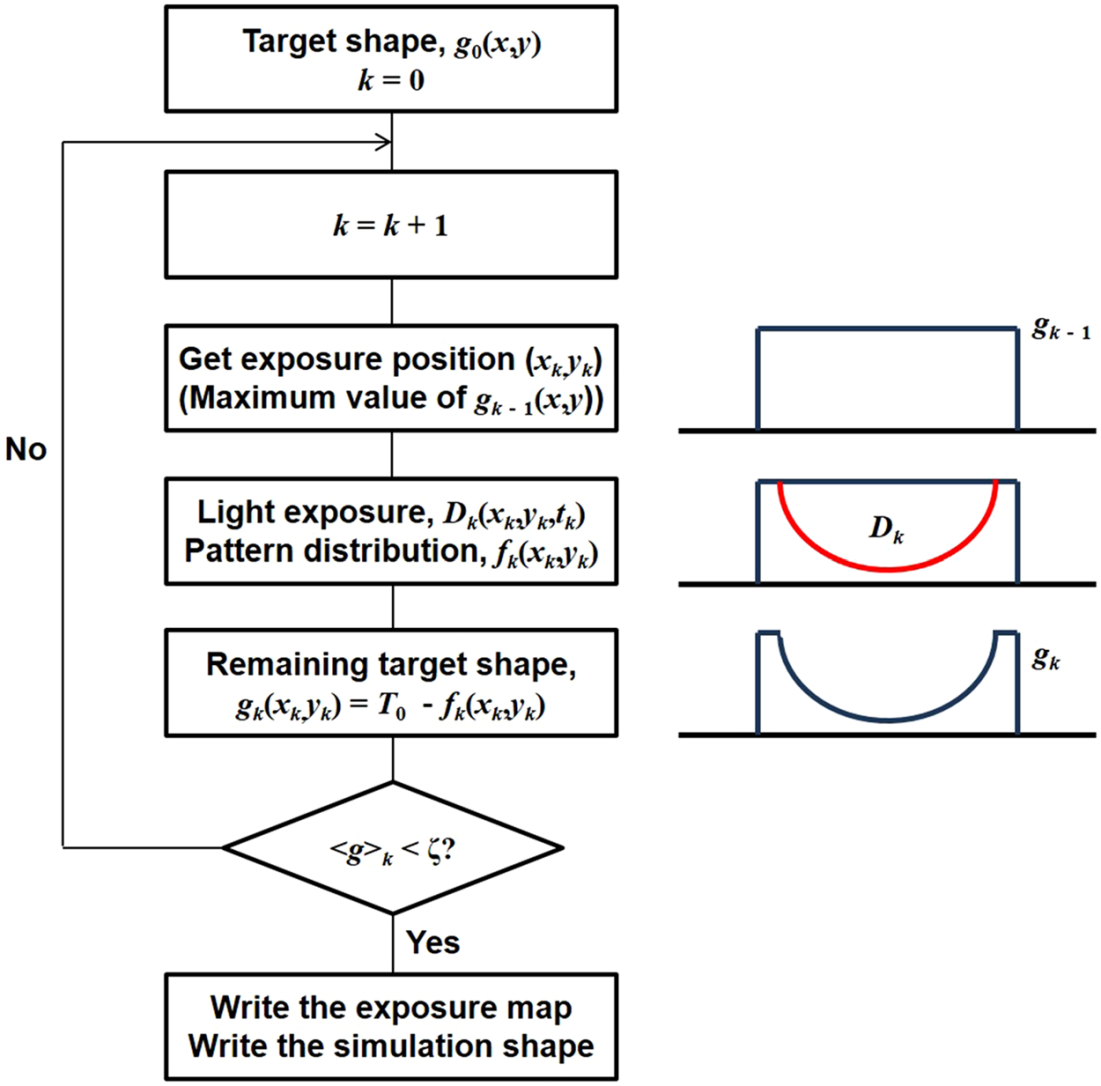
Deconvolution process for obtaining the exposure map and simulated pattern shape. Left side of figure shows the deconvolution flowchart, and right side of figure shows diagram for threshold dose line (red line) and remaining photoresist (blue line). After the *D*_k_th exposure, the region that received energy from light exposure is broadened, and the *k*th remaining target shape (*g*_k_) is changed.

Owing to the proximity of multiple exposures, the construction of delicate exposure maps and the impulse response of the system, which is the beam shape from the aperture, is required. An exposure map is directly related to the target shape and the dose distribution generated by the ridge aperture. Once the target shape is determined, it can be expressed as the convolution of the beam shape and the exposure map as follows:

2 $${{g}}_{0}(x,y)=\int D(x-{x}_{0},y-{y}_{0},t)\cdot M({x}_{0},{y}_{0}){\rm{d}}{x}_{0}{\rm{d}}{y}_{0},$$

where *M* represents the exposure map; *D* is the dose distribution, which is the impulse response of the plasmonic lithography system; and *g*_0_ represents the target depth distribution. By applying the following deconvolution technique to equation (2), an exposure map for a particular target shape can be obtained. Figure 2 shows a flowchart of the deconvolution technique used to obtain the exposure map in this work.

The deconvolution procedure used in this study requires iterations of multiple beam shapes to construct a target shape. In order to perform the deconvolution numerically, we meshed the *x* and *y* coordinates in the target shape with a mesh size of Δ*x* = Δ*y* = 20 nm. Before exposure, the target shape is defined as *T*_0_ − *g*_0_(*x*, *y*), where the subscript ‘0’ means that there is no exposure on the PR, *T*_0_ is the PR thickness, and *g*_0_(*x*, *y*) is the target depth distribution, which will be removed by beam exposure. At the position (*x*_1_, *y*_1_), where *g*_0_(*x*, *y*) is a maximum value, the first beam exposure causes an exposure dose distribution of *D*_ex_(*x* − *x*_1_, *y* − *y*_1_, *t*_1_) on the PR, and there is a pattern distribution, *f*_1_(*x*, *y*), for which the local exposure dose is larger than the threshold dose. After the exposure, the target depth distribution is converted into *g*_0_(*x*, *y*) − *f*_1_(*x*, *y*), which is equal to *g*_1_(*x*, *y*).

The iteration algorithm is performed continuously until the expectation value of *g*_*k*_ is smaller than the limit *ζ*. The figure on the right side of the diagram shows the states of *g*_*k*_ and *D*_*k*_. The blue line represents the remaining target shape *g*_*k*_, and the red line represents the light exposure. By repeating the process to find the value of the function at the maximum depth and to impose the beam exposure process, the target depth distribution is set to converge to *ζ*, and the exposure map is constructed. Mathematically, the deconvolution algorithm at the *k*th exposure can be described as

3 $${g}_{k}(x,y)={g}_{0}(x,y)-{f}_{k}(x,y).$$

As the *k*th pattern distribution is related to the summation of the local exposure dose, *f*_*k*_ is a function of $\sum _{i=1}^{k}{D}_{i}$, and is not solely determined by *D*_*k*_. For the *k*th beam exposure, the *k*th exposure time determines the maximum depth of the removed PR. This exposure time *t*_k_ can be numerically obtained from the implicit function of time and the depth calibration curve (equation (1)). During the deconvolution process, if the maximum value of *g* ^*k−*1^ corresponds to the maximum depth of the removed PR, *f*_*k*_(*x, y*) could be larger than *g* ^*k−*1^(*x, y*) in the vicinity of the exposure location (*x*_*k*_ *, y*_*k*_), which leads to an irreparable deconvolution error. In order to avoid over-patterning, the exposure time *t*_*k*_ forces *f*_*k*_(*x, y*) to satisfy the inequality

4 $$R(\nabla {g}_{k}({x}_{k},{y}_{k}),{z}_{k})\cdot {g}_{k}({x}_{k},{y}_{k}) > {f}_{k}({x}_{k},{y}_{k}),$$

where *R* is the ratio of the maximum depth of the removed PR to *g*_*k*_(*x*_*k*_ *, y*_*k*_) to preventing over-patterning, *z*_*k*_ is the target depth for PR removal, and ∇*g*_*k*_ is the gradient of the target depth for PR removal. Near the exposure location, if the slope of *g*_*k*_ is high compared with the aspect ratio of the beam shape, *R* has a lower value to reduce deconvolution error. In addition, because the aspect ratio of the beam shape is a function of *z*_*k*_, *R* is also a function of *z*_*k*_. Numerically, we obtain the function *R* with respect to ∇*g*_*k*_ and *z*_*k*_, and it is applied to the deconvolution procedure to determine *f*_*k*_(*x*_*k*_ *, y*_*k*_). By conducting the deconvolution procedure, an exposure map consisting of the exposure location and exposure time can be obtained. For e-beam lithography, their algorithms define the slope of a defined position and adjust the beam exposure to obtain the ‘targeted slope and depth’. However, our deconvolution algorithms use the slope as an exposure dose criterion only to prevent over-patterning. Hence, the targeted surface feature is ‘depth’, without aiming to obtain a slope.

**Figure 3:**
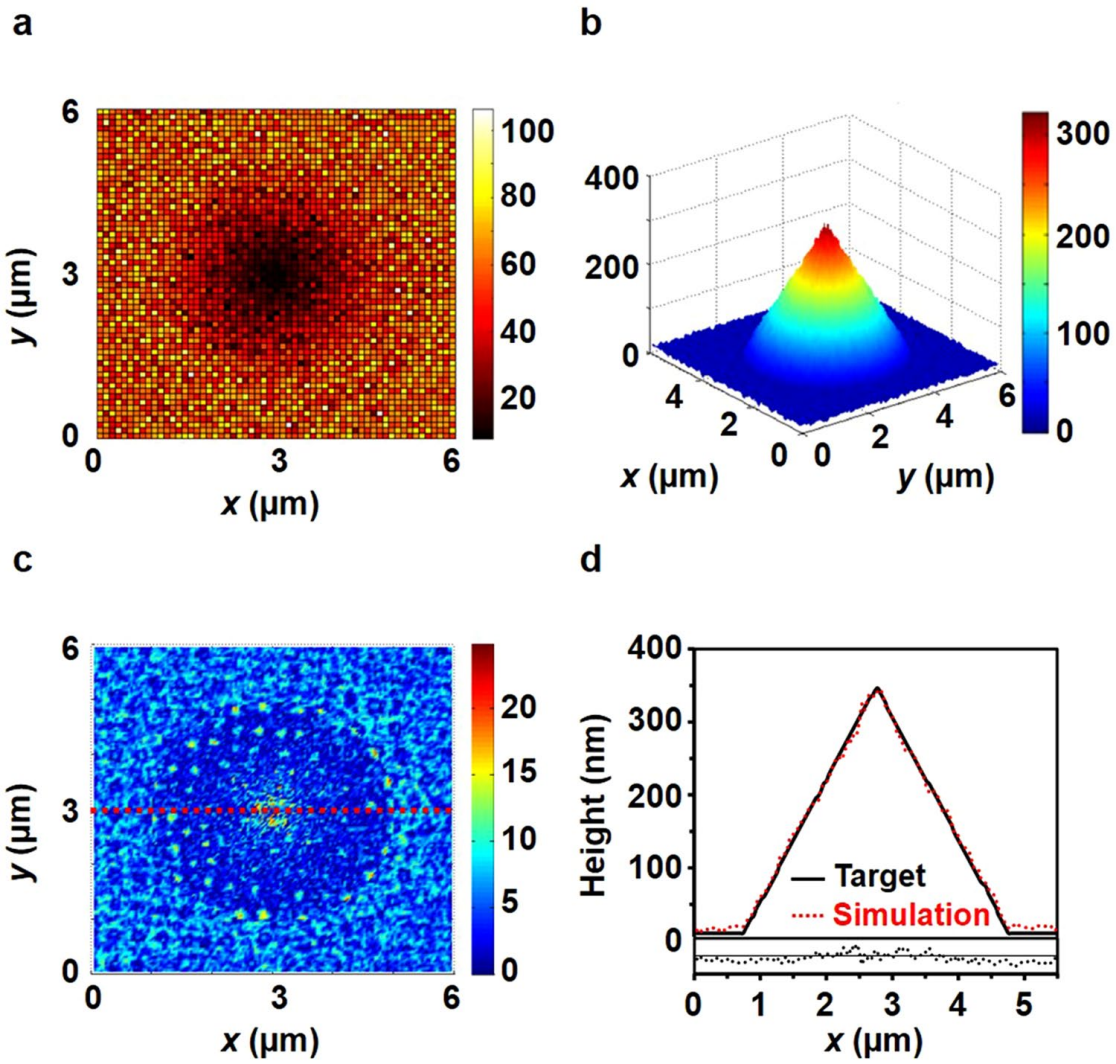
Deconvolution results. (**a**) Exposure map for a cone-shaped target pattern with a diameter of 4 µm and a height of 350 nm. To visualise the map of the exposure time (ms), each pixel shows the exposure time for areas measuring 100 nm × 100 nm, which is the result of the summation of the adjacent 5 × 5 pixels. (**b**) Simulated result of expected cone shape. (**c**) Error distribution compared with the target shape. (**d**) Profile comparison and residual error with the target shape at the centre line of the cone.

Figure 3a shows the exposure map for a cone-shaped target pattern with a diameter of 4 µm and a height of 350 nm. The cone-shaped patterning was carried out to evaluate the accuracy of the exposure map. The cone shape was selected because it is symmetrical and has an altitude that varies from 0 to 350 nm. It was necessary to confirm through the deconvolution that every direction could be fabricated uniformly, even though the spot did not have a symmetrical or atypical shape. After deconvolution, the simulated pattern shape was compared with the target shape. Figure 3b shows the simulated result of the expected cone shape. Figure 3c shows the error distribution when the simulated shape was compared with the target shape, where the root-mean-square (RMS) and P–V errors of the simulated pattern are smaller than 6 and 25 nm, respectively. The disparity between the target shape and the simulated shape is mainly determined by the number of iterations in the deconvolution process and the geometrical shape of the PSF by the ridge aperture. The accuracy can be enhanced by increasing the spot numbers but is inversely proportional to the throughput of the system; our work is therefore limited by 50,000 spots. Furthermore, because the near-field components have wide field distributions in the x- and y-dimensions and a shallow field distribution in the z-direction, there are geometrical limitations for numerical matching. To compare the simulated cone shape with the pattern results, we measure the profile of the pattern with an atomic force microscope (AFM). Before measuring the samples with AFM, we calibrated the measurement characteristic of the AFM probe with a reference sample and determined that the estimated uncertainty is less than 1 nm. Figure 3d shows the result after matching the profile of the target shape with that of the developed pattern shape; an RMS residual error of only 5.7 nm was obtained. In the modelling process, because the PSF modelling is experimentally calibrated with the geometrical dimensions of the dose-modulated spot patterns recorded on the PR, the effects of PR bleaching, change in absorptivity with light exposure, could be suppressed, which yields little disparity between the simulated shape and the patterning results.

### Multiscale 2.5D surface patterning of micro–nano structures

**Figure 4:**
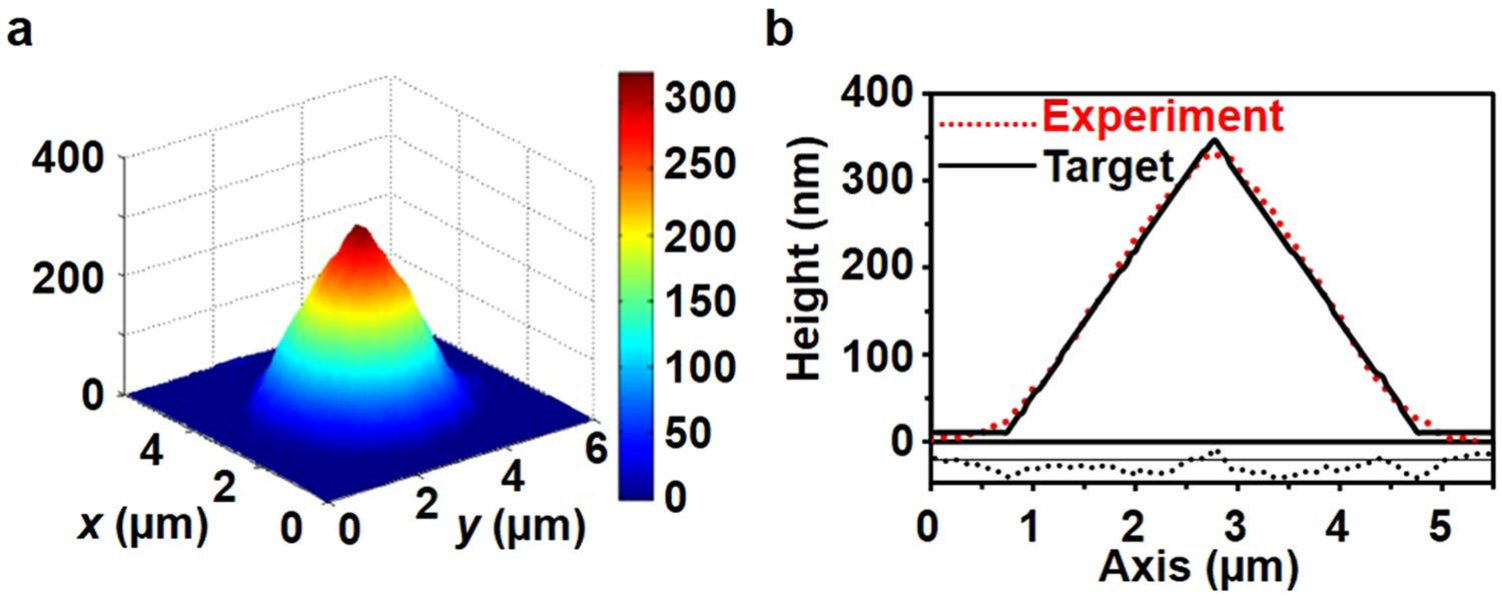
Patterning results of a cone shape. (**a**) Experimental results obtained by exposure map in Fig. 3a,b Profile comparison at the centre line between the target shape and experimental results for the cone-shaped pattern. In order to compare Fig. 4a with Fig. 3b in the same format, we plot Fig. 4a by using MATLAB.

In order to demonstrate that the patterning results are in good agreement with the target shape, we fabricated a cone-shaped pattern by using an exposure map generated by the deconvolution algorithm (Fig. 3a). In order to perform 2.5D surface patterning, we fixed the optical power as 300 μW, and the exposure time ranged from 1 ms to 100 ms. Figure 4 shows the patterning results of the cone shape and the target shape. To assess the error between the simulated shape and the pattern shape and the error between the pattern shape and the target shape, we used the height difference of the shapes in two-dimensional (*x*, *y*) points. For the error between the simulated shape and the pattern shape, we obtained an RMS error of 10.25 nm and a maximum error of 47.65 nm. In addition, an RMS error of 4.7 nm and a maximum error of 17 nm were obtained between the target shape and the pattern shape.

**Figure 5:**
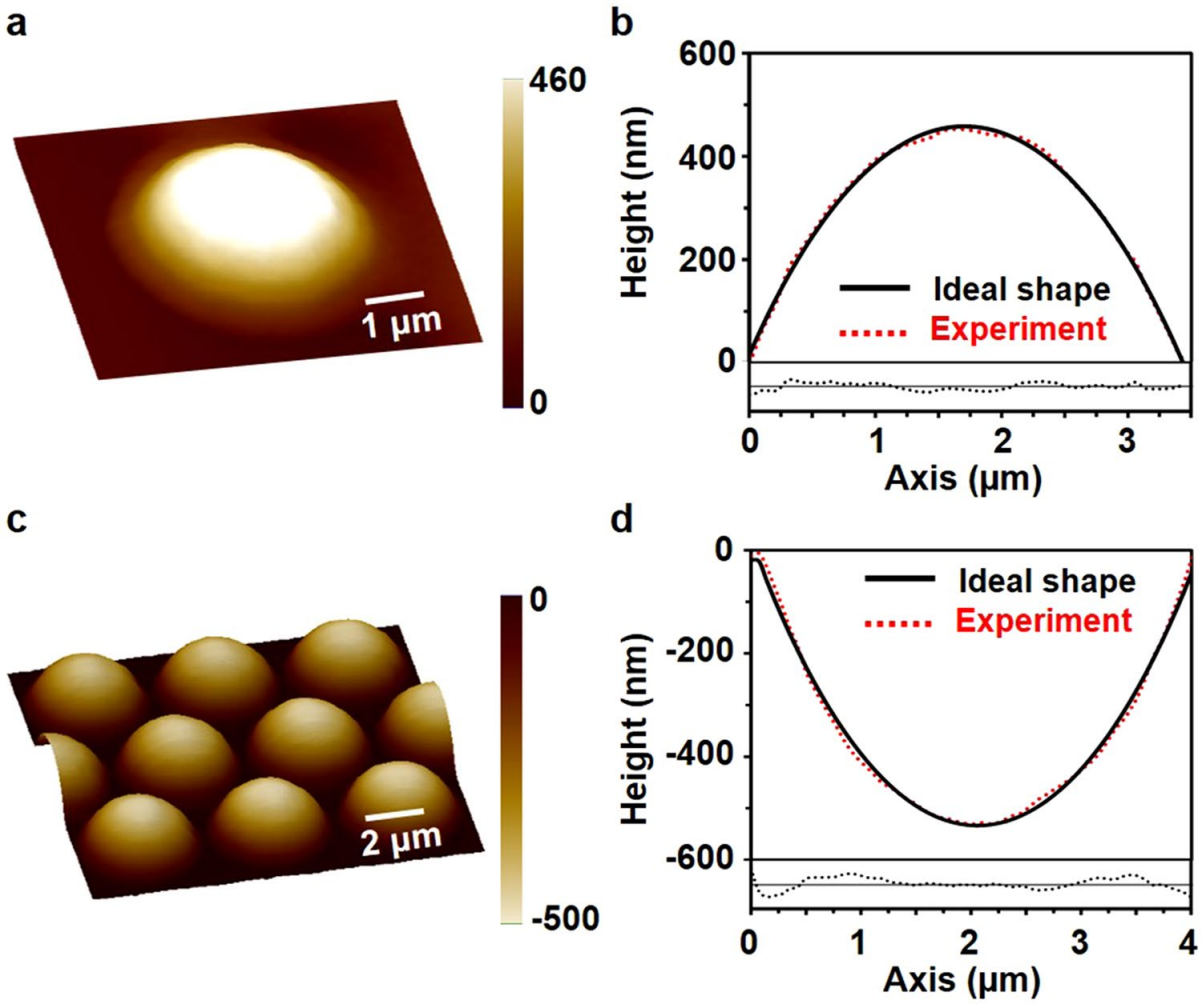
Experimental results. (**a**) Fabrication result of a single convex microlens with a diameter of 3.5 µm and height of 460 nm. (**b**) Comparison of profile of the single convex microlens with that of an ideally shaped lens having radius of curvature of 3.5 µm; residual error distribution at the centre line between the experimental result and the ideal shape. (**c**) Fabrication result of concave MLA with a diameter of 4 µm and height of 500 nm. (**d**) Comparison of profile of concave MLA with that of the ideally shaped lens having a radius of curvature of 4 µm; residual error distribution at the centre line between the experimental result and the ideal shape. the scale factor, defined as the ratio of horizontal to vertical scales, is 3 for both microlens and MLA.

**Figure 6:**
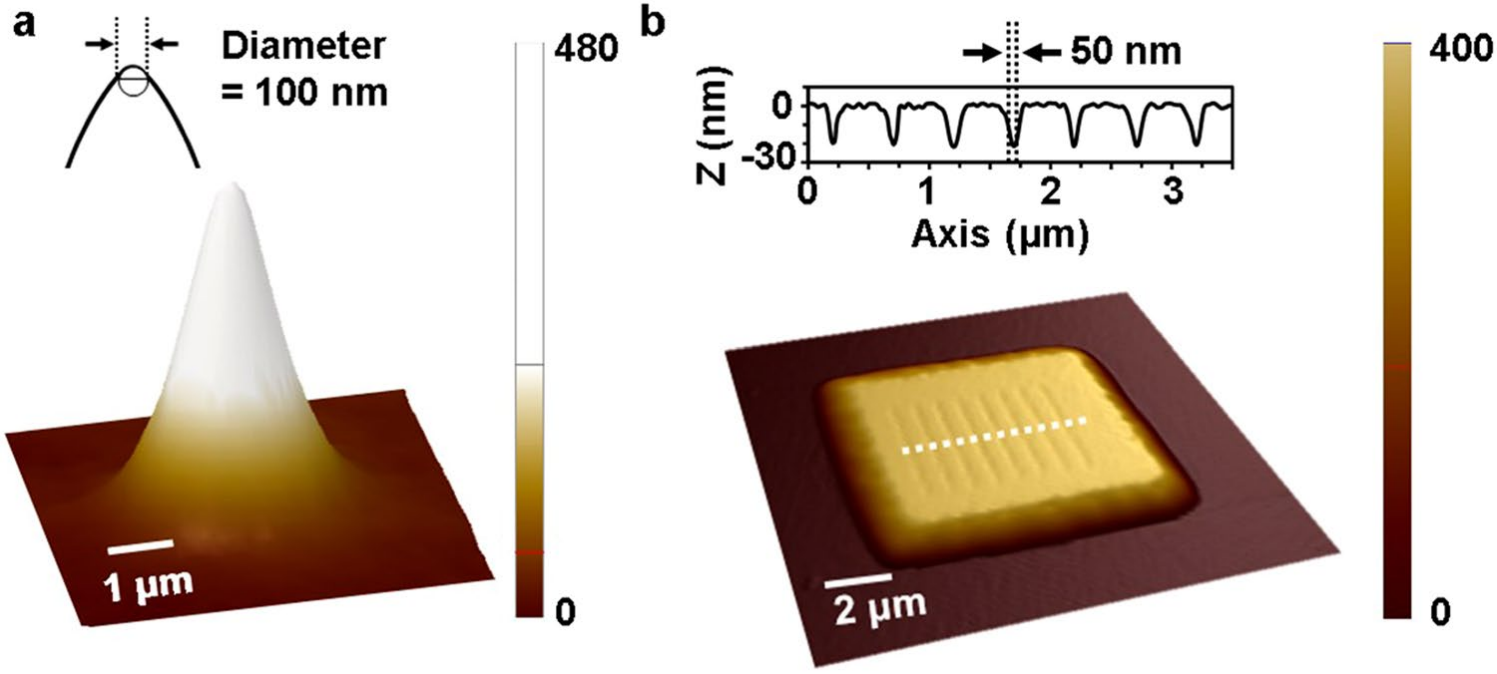
Experimental results. (**a**) Fabrication result of needle-like structure. The scale factor for needle-like structure is 7 (**b**) High-resolution line arrays of flat-top structures on the surface, visualized with realistic scale The line arrays show a 50 nm FWHM line width, which is a quarter wavelength, and a feature depth of 20 nm. The scale factor for the AFM image is 1.

We demonstrated the ability to fabricate 2.5D nanostructures using plasmonic lithography by exploring practical applications of the technique. We fabricated a ‘single microlens’ with a curved geometry. Figure 5a shows the fabrication results of the single convex microlens, which had a diameter of 3.5 µm, radius of curvature of 3.5 µm, and height of 460 nm. The focal length and the numerical aperture (NA) of the lens were 7.26 µm and 0.23, respectively. The profile comparison with the ideal shape having a radius of curvature of 3.5 µm is depicted in Fig. 6b; the plot of the residual error is shown at the bottom. The curvature was uniformly controlled in the resist and well matched with that of the ideal shape. In Fig. 5b, the RMS surface roughness over 90% of the area on the microlens top surface was approximately 7.0 nm, indicating good optical smoothness of the refractive microlens.

MLAs have been widely applied, mainly in optical systems such as wavefront sensors, backlight modules, optical interconnects, optical data storage, and imaging systems^28–30^. Several methods for fabricating MLAs using e-beam, polymer, glass, and machining have been reported^9, 31–33^. The shape factors of the MLAs in our study were measured using an AFM, and values of the radius of curvature and roughness were also obtained. Figure 5c shows the fabrication results of the concave MLAs.

Figure 5d shows a comparison of the pattern profile with that of the ideal shape and the residual error distribution at the centre line between the experimental result and the ideal shape. The fill factor of an MLA is the ratio of the effective area of light through the lens to the total area, and we obtained a fill factor of 90% by arranging the lenses in a hexagonal pattern. Each lens had a diameter of 4 µm, height of 500 nm, radius of curvature of 4.07 µm, focal length of 8.14 µm, and an NA of 0.24. In order to quantitatively analyse the uniformity of the MLAs, six lenses were randomly selected to evaluate the quality of the lens. We obtained a surface roughness of 11.5 nm and a curvature non-uniformity of 2.7% for the MLAs, which indicate that the lens arrays were fabricated uniformly and accurately compared with the ideal shape. MLA structures can be obtained with various radii of curvature by plasmonic lithography. Our proposed method has a surface quality that is comparable to that of other alternative fabrication technologies such as the FIB milling process^34^.

### Needle-like structure and high-resolution groove pattern on microscale base structure

Figure 6a shows the patterning result of a needle-like structure with a width of 4.5 µm and a sharp tip with a height of 480 nm. This needle-like structure can be used as a nanoneedle and hydrophobic surface^35–37^. To form the sharp tip, a curved side wall structure was fabricated together with a 100-nm-diameter needle. Next, we fabricated a high-resolution pattern consisting of a microscale base structure with a frustum of pyramid. Figure 6b shows high-resolution line arrays with frustum-of-pyramid structures on the top surface. The base width was 7 µm, and the flat top had a width of 5 µm and a height of 400 nm. At the top surface, sub-50-nm FWHM line arrays with a 20-nm depth were created uniformly on the top surface. We analysed the topographical profiles of the line array from the AFM data. The line edge roughness (LER) was evaluated, and a 3*σ* LER value of approximately 15 nm was obtained. By showing the frustum of the pyramid with resolutions varying from a few tens of nanometres to a few micrometres, we verified the possibility of scalable 2.5D structuring with plasmonic lithography.

As the required dimensions of nano devices vary for different purposes, scalable and cost-effective patterning is required. In addition to the potential application to scalable surface structuring, multiscale patterning by plasmonic lithography can also be applied to archive patterning, which records the pattern on a historical recording medium by engraving analogue shapes; this archived pattern is accessible at any time without the concerns of bit rot (data degradation) in digital recording. In addition, the combination of a propagating field and an evanescent field enables a multiscale spot size to be attained by varying the light intensity or exposure time, and it provides scalable depth on the micrometre scale with high volumetric throughput. By using plasmonic lithography, we demonstrated fabrication of various 2.5D structures with features with dimensions of nanometres to micrometres, thus offering new possibilities in micro/nano multi-scale structuring. With respect to the height of the structure, the height fabricated by plasmonic lithography can reach a few microns. The propagating field spreads over the space with a spherical form, which is a point source created by the bowtie aperture. The height of the structure is mainly determined by the propagating field falling off as 1/*z* ^2^, where *z* is the depth of the structure. With respect to the height of the structure, the height fabricated by plasmonic lithography can reach a few microns^16, 24^. Further, even though patterns are recorded on the PR in this paper, patterns can be transferred onto silicon with a multi-layer transfer process with a higher aspect ratio.

In summary, we used a metal ridge aperture to demonstrate nanoscale 2.5D surface patterning with plasmonic lithography. In order to fabricate the surface patterning, we used a theoretical model to describe the optical spot by a bowtie aperture in the reference. By setting the simulated spot shape as an impulse response of the plasmonic lithography system, we performed deconvolution on the spot pattern to obtain an exposure map that consists of the exposure location and exposure time. To evaluate the performance of the plasmonic lithography system, we fabricated several structures, including an MLA, a nanoneedle, and a multiscale structure. We verified the possibility of applying plasmonic lithography to multiscale structuring from a few tens of nanometres to a few micrometres in the lateral dimension. In practice, the aspect ratio of the pattern is limited by the geometrical dimensions of the PSF. As the spot size becomes larger, the aspect ratio of the PSF from the ridge aperture increases but is asymptotically limited by 0.5. We obtained an RMS error of 4.7 nm between the target shape and patterned shape and a surface roughness of 11.5 nm. We expect that the capability to generate patterns including high-resolution and microscale features at once could be applied to desktop nanofabrication with a simple optical setup and silicon-based nano/microfabrication using a PR. Furthermore, we expect the surface patterning with plasmonic lithography to facilitate fabrication of nanoscale and microscale devices.

## Methods

### Experimental system

A detailed description of the plasmonic lithography system used in this work is provided in our previous work; therefore, only a brief description is given here^17^. A circular probe with a bowtie-shaped nano-aperture on the tip was combined with a conventional laser direct-writing system. A 405 nm continuous-wave laser was used for the exposure, and an objective lens (CFI LU Plan Epi El WE, Nikon; 100 × , NA: 0.8) focused the laser beam onto the aperture. A probe made of silicon nitride (Si_3_N_4_) was coated with a 150-nm aluminium layer, and the bowtie-shaped nano-aperture was fabricated on the tip (outline dimensions: 150 nm × 150 nm; ridge gap: 25 nm) by the FIB. When the focused laser illuminated the aperture, a confined spot was formed on the exit plane, and it was used to expose the resist to form a 2.5D pattern. The probe surface was coated with a diamond-like carbon (DLC) thin film (5 nm) to minimise friction and protect the probe surface.

### Calibration of spot pattern with the analytical formula

From the AFM image, we extracted geometrical parameters with respect to the exposure dose and constructed the impulse response by plasmonic lithography. From the calibration procedure and the fitting equation, we obtain the functions *w*_*x*_, *w*_*y*_, and the coefficients *I*_surf_, *I*_space_. The *y*-width can be described by *w*_*y*_ = *f*(*D*_ex_) = 2 $\sqrt{Ct/{D}_{{\rm{th}}}}$ with *C*/*D*_th_ = 272.75 m^2^/s. The *x*-width has a mathematically complex form, so that it can be taken by the inverse function of *D*_ex_ = *g*(*w*_*x*_), which is described with *I*_surf_(*ρ, φ, 0*) by the expression

$$t={D}_{{\rm{th}}}/({A}_{{\rm{spp}}}{B}_{{\rm{spp}}}/2{w}_{x}+cos{\rm{\Phi }}/2{{w}_{x}}^{q+0.5}({A}_{{\rm{QSW}}}{B}_{{\rm{spp}}}-{A}_{{\rm{SPP}}}{B}_{{\rm{QSW}}}q/{w}_{x})+cos{\rm{\Phi }}/2{{w}_{x}}^{q+0.5}({k}_{0}{A}_{{\rm{SPP}}}{B}_{{\rm{QSW}}})-{A}_{{\rm{QSW}}}{B}_{{\rm{QSW}}}q/2{{w}_{x}}^{2q}q/{w}_{x}).$$

A full description is reported in detail in the previous work^24^. By using the parameters *q* = 1.18 and *Φ* = −0.56 calculated from the FDTD simulation results and the fitting process with equation *D*_ex_ = *g*(*w*_*x*_), we obtained *A*_SPP_ = 4.3, *B*_spp_ = −11.52, *A*_QSW_ = −1.64, and *B*_QSW_ =1.78 × 10^−4^. To obtain the 3D dose distribution, a calibration for the depth at the centre of the spot pattern is required. Because there is no metallic surface at the centre of the aperture, surface waves cannot exist, and the depth is determined by the space wave in equation (3). By substituting 0 for *ρ* in equation (3), the depth calibration curve can be expressed as *z*_*d*_ =$\sqrt{At/{D}_{{\rm{th}}}}$, where *A* is a constant related to the obtained input intensity *A*/*D*_th_ of 1588.82 m^2^/s.

### Data availability

All data generated or analysed during this study are included in this published article
